# Assessment of GHG emissions in dairy production systems based on existing feed resources through the *GLEAM* model under different climatic zones of Bangladesh and their mitigation options

**DOI:** 10.5455/javar.2024.k816

**Published:** 2024-09-29

**Authors:** Muhammad Khairul Bashar, Nathu Ram Sarker, Nasrin Sultana, Sheikh Mohammad Jahangir Hossain

**Affiliations:** 1 Bangladesh Livestock Research Institute (BLRI), Savar, Bangladesh; 2 Krishi Gobeshona Foundation (KGS), Dhaka, Bangladesh

**Keywords:** Dairy farming, different climatic zones, GLEAM-I, GHG emission, mitigation

## Abstract

**Objective:**

The current study evaluated the greenhouse gas (GHG) emissions of dairy cattle through the Global Livestock Environmental Assessment Model* (GLEAM)* model and illustrated potential mitigation strategies by modifying nutrition interventions.

**Materials and Methods:**

A semi-structural questionnaire was developed to calculate dairy animal GHG emissions. This study comprised 40 farmers from four districts: river basin (Pabna), drought-prone (Chapainobabganj), floodplain (Nilphamari), and saline-prone (Sathkhira) areas. Ten lactating cows (two cows from each farmer) were also selected to collect information on feeding practices, feed resources, feed intake (roughages and concentrate), water intake, and productive and reproductive parameters for 7 days at each site during two seasons: dry (November–February) and wet (June–October).

**Results:**

The GHG emissions from the river basin area were significantly (*p* < 0.05) higher due to low-quality roughages (75%), whereas CH_4_/kg of milk production was the lowest (77.0 gm). In contrast, the area that frequently experiences drought showed a different pattern. For instance, the generation of CH_4_ from enteric fermentation was 1187.4 tons/year, while the production of CH_4_ and N_2_O from manure management was 323.1 tons/year and 4.86 tons/year, respectively. In comparison to other climatic areas, these values were the lowest because the supply of green grass was twice as abundant as in the other climatic areas (40%). The quantity of CH_4_/kg of milk produced in an area susceptible to drought did not vary.

**Conclusion:**

Implementing feeding systems in drought-prone areas is a successful approach to reducing GHG emissions in the dairy industry in Bangladesh. Consequently, implementing feed-balancing techniques can enhance productivity and foster environmentally sustainable animal production.

## Introduction

The rising demand from emerging low- and middle-class people worldwide will lead to higher incomes with diver’s products. By 2050, there is expected to be a rise of 70% in the global demand for livestock-related items [1]. In this sense, livestock and animal-derived foods will have a crucial role in fulfilling the increasing demand for animal-based products, enhancing livelihoods, alleviating poverty, improving food security, boosting health and nutrition, and promoting gender equality [2,3].

In Bangladesh, the production of milk has increased by a factor of 4.50 (from 23.7 lakh MT to 106.8 lakh MT) between 2009–10 and 2019–20, which needed to be increased. Similarly, meat production has risen by a factor of 6.09 (from 12.6 lakh MT to 76.74 lakh MT) during the same period [4]. Two difficulties currently confront the livestock industry in Bangladesh. First, low animal productivity with poor quality roughages remains a significant problem. Consequently, poor feed resources and inadequate physiological systems are the primary causes of enhanced methane (CH_4_) emissions, resulting in the release of greenhouse gases (GHGs) and harm to the environment. The release of CH_4 _in the rumen is also an energy-inefficient process. The effect is a decrease of 6%–10% in gross energy intake or 8%–14% in digestible energy intake of ruminants [5].

In 2020, the livestock sector of Bangladesh was estimated to produce 30.1 Gg of CH_4_ (CO_2_e), whereas it emitted 26.7 Gg of CH_4_ in 2005; over 15 years, CH_4_ production gradually increased due to enteric fermentation [6]. The Paris Agreement stipulates that Nationally Determined Contributions have the objective of achieving a scenario where CO_2_ emissions are completely offset by 2050, and reducing emissions to a minimum of 43% by 2030. Addressing climate change by 2030 is a crucial objective set by the United Nations (UN) as part of the sustainable development goals (SDGs). Livestock has a significant role in addressing climate change as well as other important objectives such as poverty eradication, eliminating hunger, and promoting responsible consumption. Therefore, enhancing the digestibility of fibrous feedstuffs in ruminant animals would result in a reduction in CH_4_ emissions. This combined effect would not only minimize environmental pollution but also enhance livestock output, thereby benefiting livestock farmers. In Bangladesh, the level of GHG emissions from ruminants is low. Additionally, research activities were carried out to evaluate GHG emissions from dairy cattle using the Global Livestock Environmental Assessment Model* (GLEAM)* model and to illustrate mitigation alternatives by implementing feeding interventions.

## Materials and Methods

### Implementation location

Data used in this article were collected from four agro-climatic zones across Bangladesh [7]. These zones include the river basin area in Pabna, the drought-prone area in Chapainawabganj, the floodplain area in Nilphamari, and the saline-prone area in Satkhira district.

### Survey and data collection

A pre-validated questionnaire was developed to conduct a survey and gather results. Demographic information, livestock population, dairy cow data, key feed resources and their usage, seasonal fluctuations, production and reproduction performances, manure management, and constraints related to livestock production were gathered from specific regions. The questionnaire included closed-ended questions, which required a simple “yes” or “no” response, as well as open-ended questions that allowed for single or multiple responses.

According to the survey data, a total of 10 farmers were chosen, with each farmer having a minimum of five lactating cows from each area. The data was collected by conducting direct interviews with four specialists who were also part of the responders. Consequently, the household survey encompassed a collective of 40 farmers.

In order to get comprehensive data on dairy cows, ten crossbreed dairy cows from each site were selected, with two animals chosen from five different producers. Information regarding current feeding procedures, available feed supplies, intake of roughages and concentrate, as well as productive and reproductive data were collected from each farmer and site over seven days. Data was collected throughout two distinct seasons: the dry season (November–February) and the wet season (June–October) ranging from January 2019 to December 2019.

***Calculation of ME and crude protein (CP) requirement***

Through the utilization of the provided equation, we have computed the metabolizable energy (ME) and CP values for dairy animals, adhering to the ARC standard established in 1980 [8].

***ME requirement***

Requirement of ME for maintenance of body (MEm); MEm (MJ/day) = 8.3 + 0.091BW

Requirement of ME for milk production (MEp); MEp (MJ/kg milk) = 1.694 x EV_l_

EV_l_ = Energy value of milk = (0.0386 BF + 0.0205 solid-not-fat (SNF)—0.236 MJ/kg)

Total ME (MJ/day) = MEm + MEp

Here, BW; Body weight, BF; Butterfat (gm/kg), and SNF; Solids not fat (gm/kg)

***Calculation of CP required***

Total CP (gm/day) = RDP + UDP

RDP (gm/day) = 7.80 X ME

UDP (gm/day) = 1.91 TP—6.25 ME

TP (gm/day) = EUP + DP + MP

EUP = 6.25 (5.9206 × log_10_ BW—6.76)

DP = 6.25 (0.018 × BW^0.75^)

MP = 35.0 X milk yield,

Here, RDP; Rumen degradable protein, UDP; Rumen degradable protein, TP; Requirement of tissue protein; EUP; Endogenous urinary loss of protein, DP; Dermal loss of protein, MP; Loss of protein through milk secretion.

### Feed intake and digestibility

To assess feed intake and digestibility, we conducted a 10-day metabolic study at each location. This trial consisted of a 3-day adjustment period with a new system, followed by 7 days of data collection. Farmer strategies have been implemented without providing any additional feed to the experimental animals. The daily feed consumption was determined using the conventional method of subtracting the amount of feed remaining from the amount of feed delivered. Before the designated feeding time, the feeds were accurately measured and stored in the feed trough. The following day, the remaining feed (leftover) was collected and weighed individually. The weights were then recorded in the data collecting sheet before providing the animals with fresh feed. The dung expelled by each cow was also collected in a big bucket for 24 h and covered with a lid to prevent evaporation. Daily, the weight of cow dung in each container was measured and well-blended. Before the container was emptied, 500 gm of mixed feces was taken, and this sample was stored in a freezer at −18°C. The feces samples obtained from each cow were pooled together, and a representative sample weighing approximately 300 gm was picked for proximate analysis.

### Measurement of quantity and quality of milk

Hand milking was done two times per day, once in the morning and once in the evening. The milk production per cow was measured and recorded at regular intervals. A milk sample bottle was used to store 100 ml of milk, which was immediately placed in a refrigerator and kept at a temperature of 4°C. A pooled sample of cow milk was created by combining multiple milk samples. This pooled sample was then analyzed for milk composition at the BLRI dairy laboratory using the lacto scan analyser from Bulgaria. The equation FCM = 0.4M + 15.0F was used to calculate the 4% fat-corrected milk (FCM). In this equation, M represents milk production and F represents fat yield.

### Estimate the enteric CH_4 _emissions through the GLEAM model

The *GLEAM* represents the bio-physical processes and activities along livestock production chains under a life cycle assessment approach [9,10].

### Model description—structure and modules

The* GLEAM* is composed of five primary modules: heard, manure, feed, system, and allocation. Additionally, two supplementary modules are used to calculate direct and indirect on-farm energy use and post-farm impacts. The enteric CH_4_ emissions were estimated using the *GLEAM*-i model, which took into account the region available in the selected areas. Primary data, including intake, feed type, total dry matter intake (DMI), and productive and reproductive parameters, were obtained. In addition to the aforementioned primary data, secondary data (such as temperature, humidity, and cattle population) must be gathered to support the *GLEAM*-i model.

### Data collation and statistical analysis

The household data obtained from a questionnaire survey were summarised and analyzed using descriptive statistics, including measures such as mean, percentage, and frequency. The *GLEAM*-i model was used to estimate greenhouse gas (GHGM) emissions across four locations in livestock production systems. Data from five modules of livestock production were entered into the model. The GHG emission data from each location of the production system were evaluated using the Analysis of Variance procedure in Statistical Software for Social Science (SPSS) version 20.

## Results

### General information on dairy herd

The cattle herd size was smaller (*p* < 0.05) in the floodplain area (9 cattle/farm) compared to herds in other areas (22–29 cattle/farm) (Table 1). Similarly, milking cow herd sizes were smaller (4 cows/farm; *p* < 0.05) in the floodplain area compared to other areas (13–14 cows/farm). The conception rate (42%–56%), mortality rate (9%–11%), birth weight of calf (23–28 kg/calf), and age of 1^st^ heat (16–20 months) were similar (*p* > 0.05) across the different climatic zones (Table 1).

### Feed ration in different climate zones

**Figure 1.**
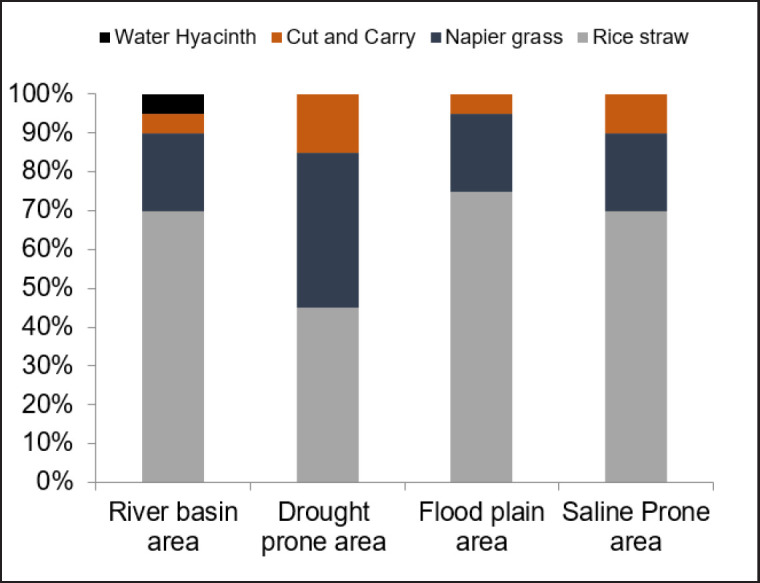
Available roughage in different climatic zones.

The farmer’s adoption of roughage feeding and the precise quantity used are depicted in Figure 1. Consumption of rice straw peaked in the floodplain region and dropped to its lowest point in the drought-prone region, with percentages of 75% and 45% correspondingly (*p* < 0.05). Nevertheless, the farmers in the zone susceptible to drought made the largest contribution of Napier grass and cut and carry grass, accounting for 40% and 15%, respectively. That is double the amount generated in other climate regions.

**Figure 2.**
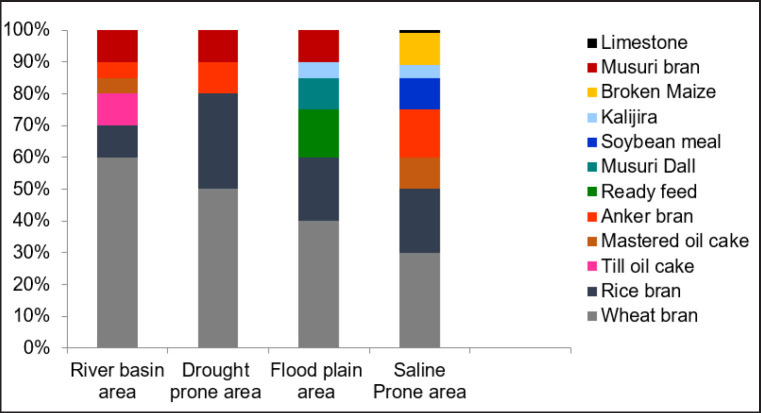
Available concentrate items in different climatic zones.

Figure 2 illustrates the farmer’s utilization of various concentrated mixed commodities in various climatic zones. The dairy cows in the river basin area consumed the most wheat bran, with a consumption rate of 60%. In contrast, the dairy cows in the saline-prone area received the least amount of wheat bran, at 30%. Additionally, it was demonstrated that the proportion of wheat bran supply increased by 10% as it transitioned from saline-prone regions to river basins. Rice bran was a common feed ingredient in all climatic zones; however, drought-prone regions utilized the highest percentage of rice bran, which was 30%. The feed ingredients that were applied in the most quantity were broken maize, limestone, mustard oil cake, Anker bran, and soybean meal, due to the saline-prone area.

**Table 1.** General information on dairy herds under different climate zones of Bangladesh.

Parameters	Different climatic zones				Significance	
	River basin area	Drought prone area	Flood plain area	Saline Prone area	SEM	Level
Herd size (no. of cattle)	26.2^ab^	28.8^a^	9.2^b^	21.6^ab^	3.19	*p < *0.05
No. of milking cows	12.8^a^	14.2^a^	4.20^b^	14.8^a^	1.58	*p < *0.05
Birth weight of calves (kg)	23.4	24.8	27.0	28.4	1.12	NS
Conception rate (%)	42.0	50.0	49.0	56.0	1.96	NS
Mortality rate (%)	10.2	11.4	13.2	9.40	0.56	NS
Age at 1^st^ Heat (months)	17.8	20.4	18.2	16.0	28.5	NS

^a,b^ means with different superscripts in the same row are significantly different at (*p* < 0.05). SEM = Standard error of mean, NS = Not significance.

### Nutrient intake

Table 2 presents the feeding habits and nutrient intake of dairy cows in various climate zones of Bangladesh. There was no statistically significant difference (*p* > 0.05) in the CP and ME requirements between the treatments. The dairy cows in the river basin area exhibited the highest dry matter (DM) intake, consuming 16.5 kg/day. This intake was substantially distinct (*p* < 0.05) from the lowest DM intake observed in the floodplain area, which amounted to 12.8 kg/day. None of the different treatments led to any significant difference in the overall consumption of CP from roughage and concentrate.

**Table 2.** Nutrient intake of crossbred dairy cows under different climatic zones.

Parameters	Different climatic zones				Significance	
	River basin area	Drought prone area	Flood plain area	Saline prone area	SEM	Level
CP requirements (gm/day)	832	733	704	1,025	50.34	NS
ME requirements (MJ/kg)	76.6	69.7	65.6	88.6	3.56	NS
DMI from Rau (kg/day)	8.90	7.76	7.47	6.68	0.32	NS
DMI from Con. (kg/day)	7.59	7.65	5.39	6.41	0.38	NS
Total DMI (kg/day)	16.5^a^	15.4^ab^	12.8^b^	13.1^b^	0.55	*p < *0.05
CPI from Rau (gm/day)	483.0	405.8	440.3	288.3	28.5	NS
CPI from Con. (gm/day)	1268	1057	930	1100	66.2	NS
Total CPI (gm/day)	1751	1463	1371	1388	81.7	NS

^a,b^ means with different superscripts in the same row are significantly different at *p < *0.05.

CP = Crude protein, ME = Metabolisable energy, DMI = Dry matter intake, SEM = Standard error of mean, NS = Not significance.

**Table 3.** Comparison of GHG emission (tons/year of each climatic zone) under different climatic zones of Bangladesh.

GHG emissions	Different climatic zones				Significance	
	River basin area	Drought prone area	Flood plain area	Saline prone area	SEM	Level
Total CH_4_	22,336.3^a^	1,510.4^b^	5,693.7^ab^	2,791.0^ab^	53.1	*p < *0.05
CH_4_ from enteric fermentation	20,262.6^a^	1,187.2^b^	3,845.7^ab^	2,363.5^ab^	67.1	*p < *0.05
CH_4_ from manure management	2,073.7^a^	323.1^c^	1,848.0^b^	427.5^c^	43.1	*p < *0.05
Total CO_2 _	205,558.5^a^	38,165.7^b^	27,638.0^ab^	22,148.4^ab^	273.4	*p < *0.05
CO_2_ from feed production	204,581.8^a^	37,885.8^b^	27,066.6^ab^	21,541.0^ab^	189.3	*p < *0.05
CO_2_ from direct energy use	883.1^a^	169.9^ab^	480.5^b^	136.6^ab^	49.3	*p < *0.05
CO_2_ from indirect energy use	93.6^b^	109.9^b^	90.8^b^	470.8^a^	53.9	*p < *0.05
Total N_2_O	1,146.0^a^	110.5^c^	213.7^b^	187.4^ab^	43.1	*p < *0.05
N_2_O from crop residue and fertilization	242.5^a^	58.7^ab^	58.7^ab^	86.6^b^	34.1	*p < *0.05
N_2_O from manure application	792.1^a^	46.9^c^	141.4^b^	91.5^ab^	13.1	*p < *0.05
N_2_O manure management	111.4^a^	4.86^c^	13.6^b^	9.26^ab^	3.34	*p < *0.05

^a,b,c^ means with different superscripts in the same row are significantly different at (*p* < 0.05). GHG = Greenhouse gas, CH_4_ = Methane, CO_2_ = Carbon dioxide, N_2_O = Nitrous Oxide, SEM = Standard error of mean.

### Comparison of GHG emissions under different climatic zones

The river basin area had the highest (*p* < 0.05) total CH_4 _emissions (22,336.3 tons/year), followed by the floodplain area (5,693.7 tons/year), the saline-prone area (2,791.0 tons/year), and the drought-prone area (1,510.7 tons/year) (Table 3). Regions susceptible to drought showed reduced CH_4_ emissions as a result of enteric fermentation and manure management, in contrast to other climatic regions. The CO_2_ emission in the river basin area was substantially greater (*p* < 0.05) compared to the drought-prone area. An identical pattern was noted in both feed production and direct use of energy. Nevertheless, the CO_2_ emissions resulting from indirect energy use were greater in the saline-prone area, amounting to 470.8 tons/year, in contrast to the other climatic zones. The river basin area exhibited the highest emissions of N_2_O. Conversely, the area of drought-prone exhibited the least amount of emissions from crop residues (58.7 tons/year), as well as from manure application (46.9 tons/year) and manure management (4.86 tons/year) (*p* < 0.05).

### Production performance and CH_4_ production

Table 4 presents the production profiles and CH_4_ emissions of crossbred dairy cows in several climatic regions of Bangladesh. No statistically significant differences were seen in the mean live weight (kg), 4% FCM production (kg/day), fat yield (kg/day), and average void production (kg/day) among various climatic zones in Bangladesh. Cattle in the river basin region consumed the greatest amount of DM per 100 kg body weight, measuring 5.80, whereas cows in the saline-prone area had the lowest intake, measuring 4.65 (*p* < 0.05). An evident disparity was seen in the mean daily milk production between the zone prone to saline (11.8 kg/day) and the zones prone to drought and flood (7.74 kg/day and 7.40 kg/day, respectively), with the former exhibiting a significantly greater value (*p* < 0.05).

**Table 4.** Production performance and CH_4_ production of crossbred dairy cows (10 cows in each location) under different climatic zones of Bangladesh.

Parameters	Different climatic zones				Significance	
	River basin area	Drought prone area	Flood plain area	Saline prone area	SEM	Level
Ave. live weight (kg)	283	276	248	282	8.09	NS
DM intake (kg/100 BW)	5.80^a^	5.70^a^	5.14^ab^	4.65^b^	0.19	*p < *0.05
CP intake (gm/ W^0.75^)	25.3	22.2	21.6	20.3	1.18	NS
Average milk production (kg/day)	9.06	7.74	7.40^b^	11.8	0.67	NS
4% FCM yield (kg/day)	8.08	7.41	7.54	10.9	0.64	NS
Fat yield (kg/day)	0.29	0.28	0.30	0.41	0.15	NS
Average void production (DM; kg/day)	3.2	3.32	3.49	3.64	0.12	NS
Methane production*						
CH_4_ production (gm/day)	565^a^	527^ab^	440^b^	448^b^	18.8	*p < *0.05
CH_4_ production (gm/kg DMI)	34.2	34.2	34.3	34.3	0.01	NS
gm CH_4_/kg milk production	77.0^b^	88.0^ab^	99.0^a^	91.0^a^	0.002	*p < *0.05

^a,b^ means with different superscripts in the same row are significantly different at (*p* < 0.05). DM = Dry matter, CP = Crude protein, FCM = Fat corrected milk, CH_4_ = Methane, SEM = Standard error of mean, NS = Not significance. *Author’s calculation as per Purnomoadi et al. [37].

**Table 5.** Effect of season on enteric CH_4_ emission in dairy cows in different climatic zones of Bangladesh.

Parameters	RBA		Sig.	DPA		Sig.	FPA		Sig.	SPA		Sig.
	Wet	Dry		Wet	Dry		Wet	Dry		Wet	Dry	
Ave. live weight (kg)	283	298	**	276	285	**	248	274	**	282	282	NS
Total DM intake (kg)	16.5	13.8	*	15.4	11.1	*	12.8	8.30	*	13.1	13.5	NS
Average milk production (kg/day)	9.10	12.8	**	7.7	6.1	*	7.4	6.7	NS	11.7	12.2	NS
Methane production (estimated)												
CH_4_ production (gm/day)	565	561	NS	527	522	NS	440	441	NS	448	445	NS
CH_4_ prod. (gm/kg DMI)	34.2	34.2	NS	34.2	34.2	NS	34.3	24.3	NS	34.3	24.2	NS
gm CH4/kg milk prod.	77.0	75.0	NS	88.0	87.3	NS	99.0	97.3	NS	91.0	89.2	NS

* = < 0.05; ** = < 0.01 and NS = Non significant. RBA = River basin area, DPA = Drought prone area, FPA = Flood prone area, SPA = Saline Prone area, aCH_4_ = Methane.

Within the river basin region, the practice of raising dairy cows led to a significantly greater release of CH_4_ (565.01 gm/day) compared to the saline-prone area (448.54 gm/day) or floodplain area (440.78 gm/day), respectively (*p* < 0.05). However, the dairy cows that were grown in the floodplain area released a higher amount of CH_4_ from the feed they consumed, with 34.3 gm/kg and 99.0 gm/kg of milk produced, respectively. By comparison, the cows in the river basin area released 34.2 gm/kg and 77.0 gm/kg of milk produced, respectively. There was a significant difference (*p* < 0.05). The two remaining groups, specifically the drought-prone area and salinity-prone area, did not exert a statistically significant influence (*p* > 0.05) on the production of CH_4_.

Table 5 displays the impact of seasonal variations on enteric CH_4_ emissions in dairy cows across several climatic zones. The seasonal fluctuation had a direct impact on the average increase in live weight, overall DMI, and average daily milk production across the three distinct climates, and this impact was statistically significant (*p* < 0.05). There was no significant variation in the productivity of dairy cows in saline-prone areas over the entire season. Furthermore, it was discovered that the seasonal fluctuations in the amount of roughage and concentrate had no noteworthy effect on the daily production of CH_4_ (gm), the production of CH_4_/kg of DMI, and the production of CH_4_/kg of milk production across the different climatic zones.

**Table 6.** Milk composition of crossbred cows in different climatic zones of Bangladesh.

Milk constituents (%)	Different climatic zones				Significance	
	River basin area	Drought prone area	Flood plain area	Saline Prone area	SEM	Level
Fat	3.20	3.70	4.10	3.59	0.13	NG
Protein	3.60^b^	3.90^b^	4.05^b^	4.50^a^	0.10	*p* < 0.05
Lactose	5.37^b^	5.75^a^	5.91^a^	5.67^a^	0.07	*p* < 0.05
SnF	9.83^b^	10.6^a^	10.8^a^	9.90^b^	0.13	*p* < 0.05

^a,b^ means with different superscripts in the same row are significantly different at (*p* < 0.05). SEM = Standard error of mean, NS = Not significance.

### Quality of milk

Table 6 displays the milk constituents found in various climatic regions of Bangladesh. There was no significant difference in the fat % of milk between the treatment groups (*p* > 0.05). The levels of protein and lactose in milk from saline-prone areas were considerably greater (*p* < 0.05) compared to milk from floodplain areas (4.05%), drought-prone areas (3.90%), or river basin areas (3.60%), respectively. Nevertheless, the SNF of milk did not exhibit a significant difference between the two climatic zones characterized by drought-prone and floodplain locations. However, it did show substantial variation among the other two climatic zones.

**Figure 3.**
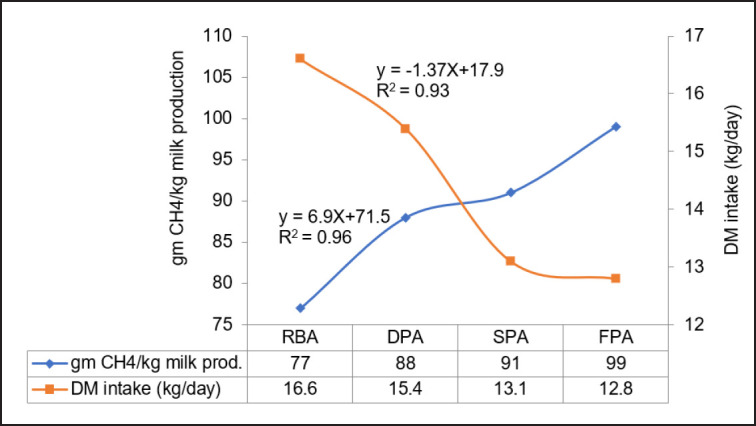
Relationship between DM intake and CH_4_ production per kg milk production.

Figure 3 displays the correlation between DMI (kg/day) and CH_4_ production per kg milk throughout different climatic zones of Bangladesh. It was noted that the CH_4_ emissions per kg of milk exhibited a linear decline (R^2^ = 0.95) when the DM intake rose. The dairy cows in the floodplain area (FPA) consumed the least amount of DM, specifically 12.8 kg/day. However, they had the highest CH_4_ emissions per kg of milk produced, which was 99.0 gm. On the other hand, the river basin area (RBA) had the lowest CH_4_ production, which was 77.0 gm, but their DM intake was the highest at 16.6 kg/day.

Given the DMI and its CH_4_ output per kg milk, the saline-prone area (SPA) appears to be a more favorable location for milk production and generating an environmentally friendly atmosphere compared to other climatic zones. Balance feed can be used as an option in many climatic zones to decrease the emission of enteric CH_4_, which can be turned into energy.

## Discussion

### Quantification of farm status and GHG emissions under different climatic zones

The global community is highly concerned with measuring and reducing the ecological consequences of the livestock industry, as it plays a significant role in the global phenomenon of climate change [11]. Estimating GHG emissions from livestock production systems is challenging; however, several methods can assist in this estimation. These tools differ in their accessibility, methodology, underlying assumptions, and range of application. These techniques are essential since they allow farmers to evaluate the sustainability of their farms and livestock production systems. This information also helps farmers in developing and implementing measures to reduce or prevent adverse consequences. The present study employed the *GLEAM* model to assess the GHG emissions originating from the livestock sector across numerous climate zones in Bangladesh.

The agriculture sector in Bangladesh released a cumulative amount of 75.0 MtCO_2_e GHG. Of the entire amount, 33% of the emissions were caused by enteric fermentation, 19% by manure, and 1% by crop residues [12], which is significantly greater than the global average of 14.5% [10]. The emissions of GHG from livestock sectors are projected to increase as a result of the rising livestock populations driven by the growing demand for animal protein, particularly in developing nations [13]. However, the reduction of CH_4_ emissions, primarily from enteric fermentation, should be achieved by 2030 to a level of 10% compared to the emissions in 2010. By 2050, the target is to further decrease CH_4_ emissions to 35% below the 2010 levels. It is necessary to decrease N_2_O emissions, primarily from fertilizers and manure, by 10% by 2030 and by 20% by 2050, within the same timeframe [14].

In the present study, the total GHG emissions from milking cows in the river basin area were significantly greater (Table 3) than in other climatic zones. This is primarily due to the substantial population of cattle and low-quality feedstuffs. On a global scale, cattle are the primary sources of emissions in the sector, responsible for around 65 percent of total emissions [10]. Moreover, a study conducted by Grossi et al. [15] has revealed that the contribution of cattle to the total GHG emissions exceeds 72%. Additionally, the river basin region exhibited a higher concentration of CH_4_ emissions from manure management (2073 tons/year) due to the utilization of liquid slurry in the river basin system, which facilitates aerobic fermentation and generates a greater quantity of CH_4_ than the open-air range/paddock systems. This is likely the result of increased utilization of concentrate feed, the predominant fodder for livestock. By contrast, drought-prone areas emitted the lowest concentration of GHG due to higher amounts of low fiber-containing green grass supplied to the animal.

Imbalance feeding of dairy cows in different climatic zones increased CH_4_ emission; floodplain areas produced the highest amount of CH_4_ (99.0 gm CH_4_/day) compared to others.

The objective of the current investigation was to intentionally disrupt the nutrient equilibrium to manipulate the rumen fermentation pattern, which led to a decrease in the number of microbial cells. The observed decrease in propionate production may have led to an increase in acetate and butyrate production, which could have increased CH_4_ emission [16]. The findings indicate that there is potential for decreasing enteric CH_4_ emissions in Bangladesh’s small-scale dairy production system, characterized by lower to moderate average productivity of dairy cows. To enhance productivity, it is possible to provide dairy cows with a carefully balanced diet that is rich in essential nutrients. Additionally, Króliczewska et al. [17] noted that by modifying the food of dairy animals, there is a significant chance to enhance milk production and lower GHG emissions from these animals. Productivity would rise and GHG emissions from animals would decrease with improved fertilizer usage efficiency. Improving feed conversion efficiency can result in higher output and a considerable decrease in CO_2_-eq emissions per kilogram of milk [18,19].

### Mitigation options

The mitigation of enteric CH_4_ is a practical approach to reducing the contribution of agricultural livestock to climate change [20]. It represents an update on enteric CH_4_ mitigation intrusions by incorporating dietary manipulation [20,21,22], which influences the composition of feedstuffs, and an inexpensive approach to forward the rumen fermentation process to reduce CH_4_ emissions [23]. It includes improving roughage quality and decreasing the relative proportion of roughage to concentrate.

**Improvement of roughage quality**

Roughage constitutes a substantial proportion of the diet for ruminant animals. Enhancing the quality and ease of digesting fibrous feed is a potential measure for reducing CH_4_ emissions [24]. Increasing the digestibility of roughage reduces the intensity of CH_4_ emissions by raising the amount of energy that animals may obtain from it. Nevertheless, cattle farming in four different climatic regions of Bangladesh provides low-quality feed, which leads to an increase in lignin production on the cell wall. This, in turn, reduces the cell wall’s degradability and polysaccharide breakdown. Consequently, the use of poor-quality roughages might lead to an increase in both the production and concentration of CH_4_ due to a higher C:N ratio and reduced digestibility.

High-grade forage has the potential to decrease CH_4_ emissions originating from the rumen. Young plants of high quality possess a greater quantity of easily fermentable carbohydrates and lower levels of neutral detergent fiber (NDF), resulting in a modification of the process of fermentation that can decrease the intensity of enteric CH_4_ emissions [24,25]. Additionally, it enhances the digestibility and passage rate. For instance, adding grass silage or herbage to low-quality roughage increased the digestibility of dairy animals by 25%. Similarly, the CH_4_ yield and intensity decreased by 10% and 19%, respectively, as a result of greater transit from the rumen and improved animal production [26].

Geographical location is another factor affecting roughage digestibility and CH_4_ production. For example, ruminant diets in temperate regions often consist of C3 grasses and cool-climate legumes, whereas C4 grasses and warm-climate legumes are utilized in tropical regions. C4 grasses exhibited a 10%–15% higher production of CH_4_ compared to C3 grasses. This can be attributed to the lower lignin and NDF content and the faster rate at which C3 grasses move through the rumen. Replacing grass silages with legume silages can potentially decrease CH_4_ emissions since legume silages have lower fiber contents and contain bioactive substances [27,28].

Commonly, the grass is harvested at a later maturity stage, resulting in reduced sugar, N, and dOM concentrations. After grass silage is made, lactate is produced in the silo and often added to the silage via an ensiling process. Subsequently, grass silage-fed animals emit higher CH_4_ than maize silage [20,21]. The reason for this is that maize silage or other small grains that comprise whole-crop silage typically have greater levels of easily digestible carbohydrates, such as starch. This leads to an increase in DMI and improves animal performance, ultimately leading to reduced CH_4_ output from the animals [29].

Three possible strategies can be performed to reduce CH_4_ emissions in the rumen when using maize or whole-crop silage. Initially, a significant amount of starch leads to the production of propionate rather than acetate. Furthermore, it augments overall DMI and the rate at which it passes through the digestive system, reduces the ruminal retention time and its fermentation, and improves post-ruminal digestion. Furthermore, substituting grass silage with maize silage leads to a decrease in CH_4_ emissions per unit of animal output, hence enhancing animal production efficiency. Hassanat et al. [30] showed a decrease in CH_4_ emissions when alfalfa hay was completely substituted with maize silage.

**Decreasing the relative proportion of roughage to concentrate**

Concentrates are frequently incorporated into feed in various quantities, as roughage alone is insufficient to improve animal performance. CH_4_ emissions went down due to changes in patterns of rumen fermentation and the proportions of Volatile fatty acids (VFAs) production that happened when the ratio of roughage to concentrate was decreased. VFAs and CH_4_ emissions also varied depending on the breakdown of various carbohydrates in the rumen. Roughage consists of complex carbohydrates, specifically cellulose and hemicellulose, that stimulate the production of acetate and butyrate. This, in turn, increases the amount of H_2_ available for methanogenesis [31].

In contrast, concentrates consist of non-structural carbohydrates, such as starch and sugar, that enhance the formation of propionate. Propionate, as a glucose and lactose precursor, uptakes H_2_ and raises metabolic energy compared to CH_4_ belching. It has been reported that adding 35% or 60% concentrate to feed reduces CH_4_ production and enhances productivity. On the other hand, studies have demonstrated that elevated levels of concentrates might stimulate the production of lactic acid and VFAs in the rumen, leading to health issues such as sub-acute ruminal acidosis [32,33]. The addition of concentrate (386 gm/kg DM) to the diets of beef cattle resulted in a 26% decrease in the synthesis of CH_4_. Similarly, the addition of concentrate to the diets of dairy cattle led to a 14% decrease in CH_4_ emissions, while in sheep, it resulted in a 6% decrease [26]. Furthermore, the quantity of dry-rolled maize added to the diet of beef steers increased from 225 to 838 gm/kg DM. As a result, there was a reduction in CH_4_ emission and an enhancement in the conversion efficiency from digestible energy to metabolizable energy [34].

The grazing ruminant diet has a better ability to raise high fermentable carbohydrate intake; however, it depends on the baseline intake of high-quality forage, which influences enteric CH_4_ emissions [35,36]. For example, when low-to-moderate quality feed for cows was enhanced with 281 gm/kg DM and 461 gm/kg DM concentrate, there was a linear rise in CH_4_ emissions, whereas CH_4_ yield and intensity declined [36]. Thus, including dietary concentrate can significantly reduce enteric CH_4 _emissions when the basal diet consists of low-quality roughages.

## Conclusion

In the end, the river basin region had the highest GHG emissions compared to other climate zones. This shows that poor inputs in the feeding system and a larger cattle population both played a role in the rise in GHG emissions. However, feed intake, CH_4_ emission reduction, and the influence of seasonal variations on the rearing system of dairy cows in drought-prone areas were deemed superior feeding practices compared to others. Implementing balanced feed in various climatic zones can potentially mitigate enteric CH_4_ emissions, which can be used as energy and ultimately enhance cattle output in Bangladesh. Furthermore, the results of this project will assist policymakers in the livestock industry in formulating future research plans with extensive efforts.

## List of Abbreviations

CP, Crude protein yield; CH_4_, Methane; DM, Dry matter; FCM, Fat-corrected milk; GHG, Greenhouse gas; NDF, Neutral detergent fiber.
